# Patterning of supported gold monolayers via chemical lift-off lithography

**DOI:** 10.3762/bjnano.8.265

**Published:** 2017-12-08

**Authors:** Liane S Slaughter, Kevin M Cheung, Sami Kaappa, Huan H Cao, Qing Yang, Thomas D Young, Andrew C Serino, Sami Malola, Jana M Olson, Stephan Link, Hannu Häkkinen, Anne M Andrews, Paul S Weiss

**Affiliations:** 1 California NanoSystems Institute, University of California, Los Angeles, Los Angeles, CA 90095, USA; 2 Department of Chemistry and Biochemistry, University of California, Los Angeles, Los Angeles, CA 90095, USA; 3 Department of Physics, Nanoscience Center, University of Jyväskylä, FI-40014 Jyväskylä, Finland; 4 Department of Materials Science and Engineering, University of California, Los Angeles, Los Angeles, CA 90095, USA; 5 Department of Chemistry, Rice University, Houston, Texas, 77005, USA; 6 Department of Electrical and Computer Engineering, Rice University, Houston, Texas, 77005, USA; 7 Department of Chemistry, Nanoscience Center, University of Jyväskylä, FI-40014 Jyväskylä, Finland; 8 Department of Psychiatry and Biobehavioral Sciences, Semel Institute for Neuroscience and Human Behavior, and Hatos Center for Neuropharmacology, University of California, Los Angeles, Los Angeles, CA 90095, USA

**Keywords:** chemical patterning, hybrid material, monolayer, soft lithography, two-dimensional material

## Abstract

The supported monolayer of Au that accompanies alkanethiolate molecules removed by polymer stamps during chemical lift-off lithography is a scarcely studied hybrid material. We show that these Au–alkanethiolate layers on poly(dimethylsiloxane) (PDMS) are transparent, functional, hybrid interfaces that can be patterned over nanometer, micrometer, and millimeter length scales. Unlike other ultrathin Au films and nanoparticles, lifted-off Au–alkanethiolate thin films lack a measurable optical signature. We therefore devised fabrication, characterization, and simulation strategies by which to interrogate the nanoscale structure, chemical functionality, stoichiometry, and spectral signature of the supported Au–thiolate layers. The patterning of these layers laterally encodes their functionality, as demonstrated by a fluorescence-based approach that relies on dye-labeled complementary DNA hybridization. Supported thin Au films can be patterned via features on PDMS stamps (controlled contact), using patterned Au substrates prior to lift-off (e.g., selective wet etching), or by patterning alkanethiols on Au substrates to be reactive in selected regions but not others (controlled reactivity). In all cases, the regions containing Au–alkanethiolate layers have a sub-nanometer apparent height, which was found to be consistent with molecular dynamics simulations that predicted the removal of no more than 1.5 Au atoms per thiol, thus presenting a monolayer-like structure.

## Introduction

Chemical lift-off lithography (CLL) is a subtractive technique for patterning self-assembled alkanethiol molecules on Au surfaces via rupture of Au–Au bonds at the Au–monolayer interface [1–2]. In CLL, hydroxyl-terminated molecules (or other species with reactive termini) in preformed self-assembled monolayers (SAMs) are lifted off Au surfaces through contact with O_2_-plasma-activated poly(dimethylsiloxane) (PDMS) stamps. Compared with microcontact or transfer printing methods [3–6], CLL produces crisp, stable patterns with sub-20 nm resolution and patterned areas of more than square millimeters [1,7]. We have used CLL on gold to control the placement and nanoscale environment around surface-immobilized biomolecules and to simplify patterning steps in device fabrication [1–2,7–13].

Two-dimensional (2D) materials have proven to be extremely rich in terms of new and potentially useful properties [14–18]. Here, we have investigated Au–alkanethiolate layers on PDMS that were produced during CLL specifically for their 2D material properties. The existence of Au on the PDMS stamp following lift-off was initially discovered using X-ray photoelectron spectroscopy (XPS) to investigate post-CLL PDMS stamps [1]. These Au layers had been predicted in a gedankenexperiment by George Whitesides, in which he described the strength of the Au–S bond as stronger than the (weakened) bond between the top layer of alkanethiolate-bound Au atoms and the underlying Au substrate. The layers removed during CLL have not yet been well characterized.

In CLL, the height difference between the remaining SAM and the contact region (where molecules were removed) was the thicknesses of the SAM plus ≈4 Å [1]. This height difference is consistent with one or at most two layers of Au being removed by CLL. Although not fully elucidated, we refer to the lifted-off species as a (supported) Au–alkanethiolate monolayer (vide infra).

Chemical lift-off lithography differs from other subtractive or deterministic transfer printing techniques [6,19–23] in that the stamp “inks” used during the transfer have a different composition than the inks originally deposited onto the substrates. While other types of thin Au films and Au nanoparticles are identified through their measurable geometry- or size-dependent optical and electronic properties (e.g., localized surface plasmons) [24–26], we show that CLL lifted-off monolayers lack significant optical signals that make them distinguishable from the PDMS supporting matrix. Using contrast methodologies, we determine that the chemistry of the supported Au monolayers remains consistent with that of bulk Au.

We used experimental and computational strategies to characterize the hybrid Au–alkanethiolate 2D material formed at PDMS surfaces via lift-off lithography. Chemical lift-off lithography was used to pattern featureless (flat) PDMS substrates with Au–alkanethiolate monolayers, which enabled direct characterization of the nanometer-scale heights of the supported Au monolayers through scanning probe microscopy, as well as the exploration of spatially encoded functionality using fluorescence microscopy. Otherwise, when topographically patterned PDMS stamps are used to pattern Au monolayers, the traits of the latter are overwhelmed by the PDMS features that are hundreds of nanometers thick. These features are indiscernible on flat PDMS without the application of patterned reference regions, i.e., regions that contain only PDMS adjacent to areas containing monolayers of Au–alkanethiolate complexes.

To gain insight into lift-off lithography removal mechanisms and outcomes of the lift-off process at the atomic scale, we simulated lift-off using molecular dynamics and density functional theory. We determined the energetics of this complex system during lift-off. The simulations were used to predict the stoichiometry and structure of the lifted-off Au–alkanethiolate monolayers. The calculated stoichiometry estimated the limits for the structure of the Au–alkanethiolate monolayers, guiding our interpretation of the existence of Au monolayers.

## Results and Discussion

**Figure 1:**
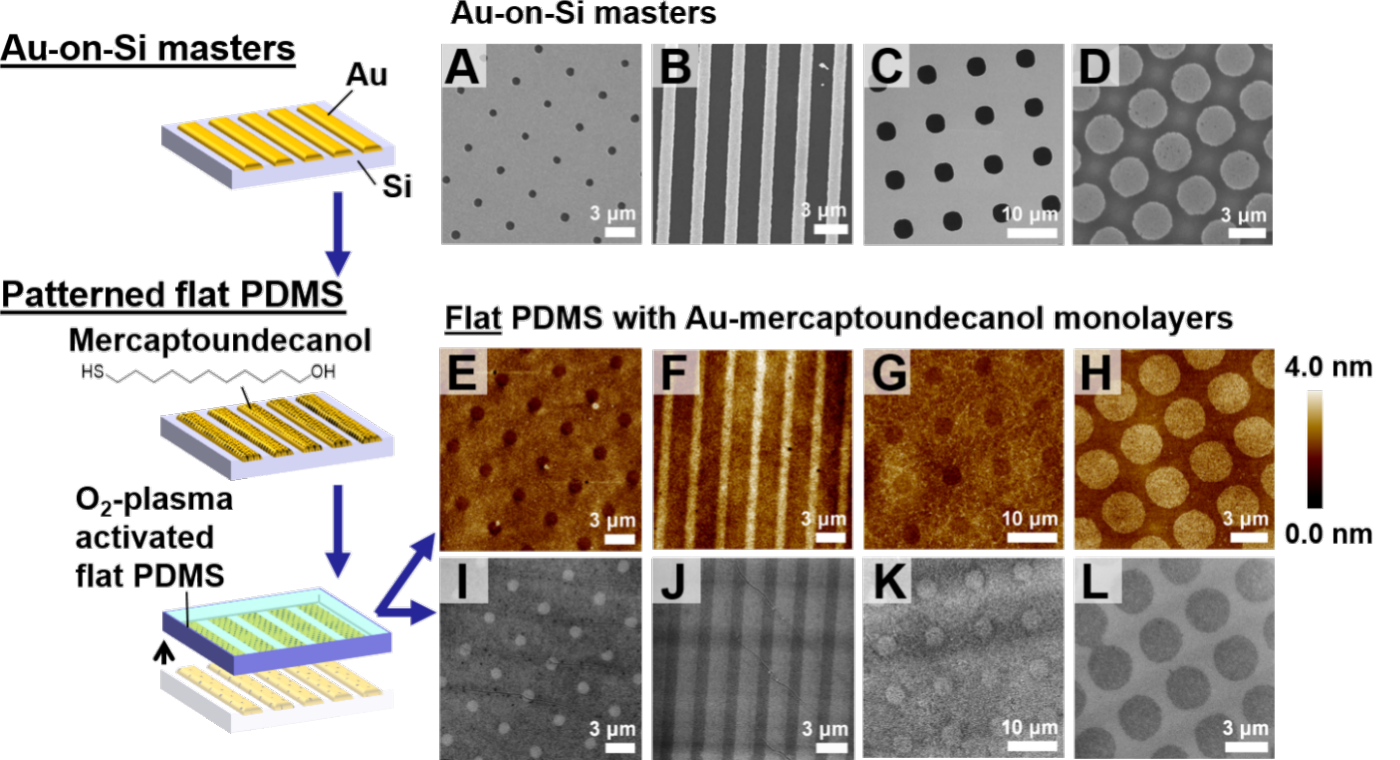
(Left) Scheme for patterning flat poly(dimethylsiloxane) (PDMS) substrates with Au–mercaptoundecanol monolayers. The Au features (100 nm height) on the Au-on-Si masters were functionalized with mercaptoundecanol and contacted with topographically flat PDMS stamps. (A–D) Scanning electron micrographs of Au-on-Si masters with (A) 1 μm diameter holes, (B) 1 μm lines, (C) 5 μm diameter holes, and (D) 3 μm diameter raised circles. The Au regions appear bright in these images. (E–H) Height maps of Au–mercaptoundecanol monolayers on PDMS produced from the Au masters in panels A–D, respectively. Images were acquired by peak-force atomic force microscopy. (I–L) Variable-pressure scanning electron micrographs of the same Au–alkanethiolate patterns on PDMS visualized in panels E–H. Images in panels I–L are contrast-enhanced to visualize the features more clearly. The original images are shown in Figure S2 (Supporting Information File 1).

Au-on-Si master substrates were patterned by a first round of CLL. Here, topographically patterned PDMS stamps were used to lift-off hydroxyl-terminated self-assembled alkanethiols (Figure S1, Supporting Information File 1) [1,9]. Following this CLL step, Au in the lifted-off (exposed) regions was removed by wet etching to form Au features in the noncontact regions. Next, hydroxyl-terminated alkanethiols were self-assembled on the patterned Au masters (Figure 1, left). Topographically flat, activated PDMS was brought into contact with the patterned Au masters to carry out a second round of CLL that resulted in otherwise featureless PDMS that was patterned only with the Au–alkanethiolate monolayers.

We imaged patterns of Au–alkanethiolate monolayers on PDMS substrates using nanoscale characterization tools. The topographies were measured using peak-force atomic force microscopy (PF-AFM), an intermittent-contact mode suitable for interrogating soft samples [27]. The AFM topography map in Figure 1E shows a pattern of recessed circular holes, which are each approximately 1 µm in diameter with a center-to-center separation of 4 µm. These features directly reproduced the lateral dimensions and periodicity of the Au features on the corresponding Au-on-Si master imaged by SEM in Figure 1A. The remaining images in Figure 1F–H demonstrate the same characteristics; the protruding regions in each AFM height map of post-lift-off PDMS corresponded directly to the raised Au features on the related Au-on-Si masters. Thus, the PDMS substrate was patterned by the addition of the Au–mercaptoundecanol monolayers from the patterned Au regions on the Au-on-Si masters, and not by imprinting, as nanoimprinting would result in inverse height topographies from those observed in Figure 1E–H. Notably, after reannealing and further self-assembly of new alkanethiol monolayers, the Au-on-Si masters could be reused a number of times to pattern multiple PDMS samples (Figure S3, Supporting Information File 1).

Patterned lifted-off monolayers were also imaged using variable-pressure scanning electron microscopy (VP-SEM), as shown in Figure 1I–L. Compared with AFM, SEM can be used to image patterns more efficiently as it provides chemical sensitivity and faster image acquisition over larger areas (up to square millimeters) [5,28–30]. The VP-SEM modality accommodates nonconducting samples by injecting water vapor into the sample chamber to offset destructive charging of the sample. In all cases, the dimensions and feature arrangement on patterned PDMS samples were consistent with those observed by AFM. In the VP-SEM images, functionalized regions consistently appeared less intense than the surrounding regions. We previously observed a similar contrast inversion while imaging self-assembled alkanethiols on Au surfaces [5]. In earlier studies, changing the operating voltage (i.e., the voltage of the primary electron beam) during SEM image acquisition was shown to reverse the contrast for images taken from the same sample. For PDMS, which is not conducting, the accelerating voltage, sample height, and vapor pressure were adjusted so that patterns could be discerned. The level of contrast in VP-SEM also depends on the nature of the alkanethiol molecules and SAM disorder (e.g., the orientation and conformation of the molecules in the SAM) [5]. The Au–mercaptoundecanol monolayers on PDMS are disordered as only 60–70% of alkanethiol molecules are removed during CLL [1,10,31]. The resulting incomplete coverage may also influence the observed contrast. These Au–alkanethiolate monolayers on PDMS are composed of Au atoms bound to the PDMS by organic alkanethiol molecules. Thus, the observed contrast of Au, as seen in the SEM images of the Au-on-Si masters (Figure 1A–D), is not necessarily comparable to that of the SEM images of Au–alkanethiolate monolayers (Figure 1I–L).

We were unable to image patterned lifted-off monolayers on PDMS using optical extinction spectroscopy (Figure S4, Supporting Information File 1). We attempted to quantify the optical extinction of lifted-off Au monolayers on PDMS using a strategy previously employed to measure extinction from assemblies of Au nanoparticles or nanometer-thin Au films [32]. As shown in Figure S4C (Supporting Information File 1), the optical extinction was indistinguishable from the instrument noise in the visible wavelength region. Furthermore, there were no discernable differences in transmission between regions containing the Au monolayers and unmodified PDMS. Therefore, the Au–alkanethiolate hybrid material is transparent at visible wavelengths to within our measurement capabilities.

**Figure 2:**
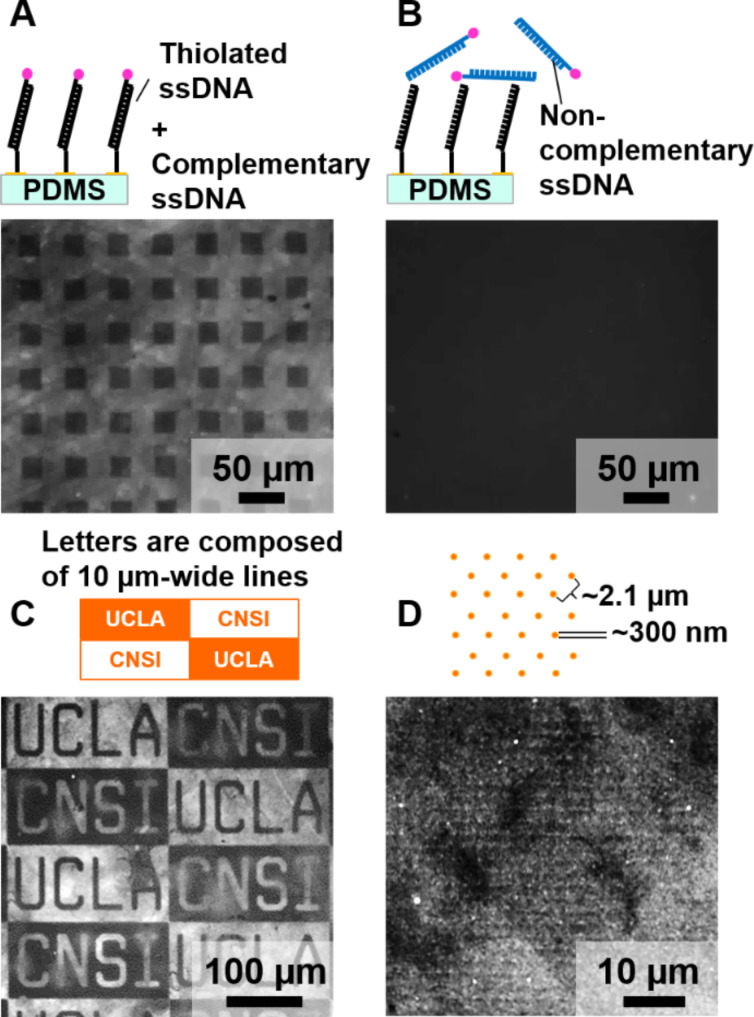
Fluorescence visualization of patterned lifted-off Au–alkanethiolate monolayers via DNA self-assembly and hybridization. (A) (Top) Scheme for complementary DNA hybridization experiments. (Bottom) Fluorescence microscopy image of a Au–alkanethiolate monolayer pattern on flat poly(dimethylsiloxane) (PDMS) after incubation with thiolated single-stranded DNA. Bright (lifted-off) regions between squares are indicative of hybridization of AlexaFluor^®^ 488-labeled complementary DNA. Square regions are dark due to the absence of Au, and therefore, also the absence of self-assembled DNA necessary for hybridization. (B) (Top) Scheme for noncomplementary control experiments. (Bottom) Similar substrate and DNA self-assembly as in panel A with the exception that scrambled, noncomplementary, fluorescently labeled DNA was used for hybridization. (C) Flat PDMS was patterned with Au–alkanethiolate monolayers in the “CNSI” lettered and “UCLA” relief regions. The patterns were then visualized using the same DNA self-assembly and hybridization procedure as in panel A. (D) A different region of the same PDMS sample shown in panel C but patterned with 300 nm dots having a nearest-neighbor center-to-center separation of 2.1 µm.

Although the Au monolayers were not optically detectable, we labeled them with thiolated DNA using a strategy to detect even minor amounts of species via their chemical properties [33–35]. In doing so, we demonstrated the chemical functionality of the Au–alkanethiolate monolayers (Figure 2). Complementary DNA was hybridized to thiolated single-stranded DNA self-assembled on lifted-off Au-containing regions on PDMS samples. Complementary sequences were fluorescently labeled, enabling indirect visualization of patterned Au monolayers. Only regions containing Au–alkanethiolates appeared bright in fluorescence microscopy images (Figure 2).

Using this straightforward functionalization and visualization method, we investigated patterns of lifted-off Au monolayers on PDMS as substrates for DNA recognition. Upon hybridization of dye-labeled complementary strands, fluorescent patterns were readily observed (Figure 2A,C,D). No measurable fluorescence was detected when DNA-functionalized substrates were exposed to dye-labeled non-complementary DNA (Figure 2B). Thus, the fluorescence patterns observed in Figure 2 derive from specific hybridization between thiolated DNA strands and their complementary sequences. Also, no patterns were observed in control experiments investigating nonspecific adsorption of complementary strands to patterned substrates in the absence of self-assembled thiolated DNA, hybridization with noncomplementary self-assembled DNA, or self-assembly and hybridization of DNA on unpatterned PDMS (Figure S5, Supporting Information File 1).

Using CLL and fluorescence imaging, we produced images over square-millimeter areas with a lateral feature size spanning several orders of magnitude on the same substrates (Figure 2C,D). We have yet to determine the limits of the feature size and area that can be patterned by CLL, where features as small as 5 nm have been removed from the original monolayer [2]. In addition to the production of a wide range of feature sizes, another important advantage is that the supported Au monolayer on PDMS samples were stable for at least six months (Figure S6, Supporting Information File 1). These results suggest that while the optical properties of the lifted-off monolayers are different from those of bulk Au (i.e., the former are optically transparent), lifted-off Au monolayers are chemically similar to bulk Au since they are amenable to self-assembly of thiols, and thus, to forming Au–S bonds. The chemical ability to modify the supported Au monolayers resulting from CLL implies opportunities for large-scale, transparent, sensor technologies, which could be straightforwardly fabricated under ambient conditions.

Having established characterization modalities to evaluate Au–alkanethiolate monolayers on PDMS, we developed an additional strategy for patterning PDMS with Au–alkanethiolate monolayers that takes advantage of the chemical selectivity associated with CLL. We previously determined that methyl-terminated SAMs do not react with activated PDMS and are therefore inert to lift-off. Terminal functional groups that are “CLL compatible” include hydroxyl, amino, carboxylate, and phosphonate moieties, such that these groups react with oxidized PDMS and are lifted off [1,10–11].

**Figure 3:**
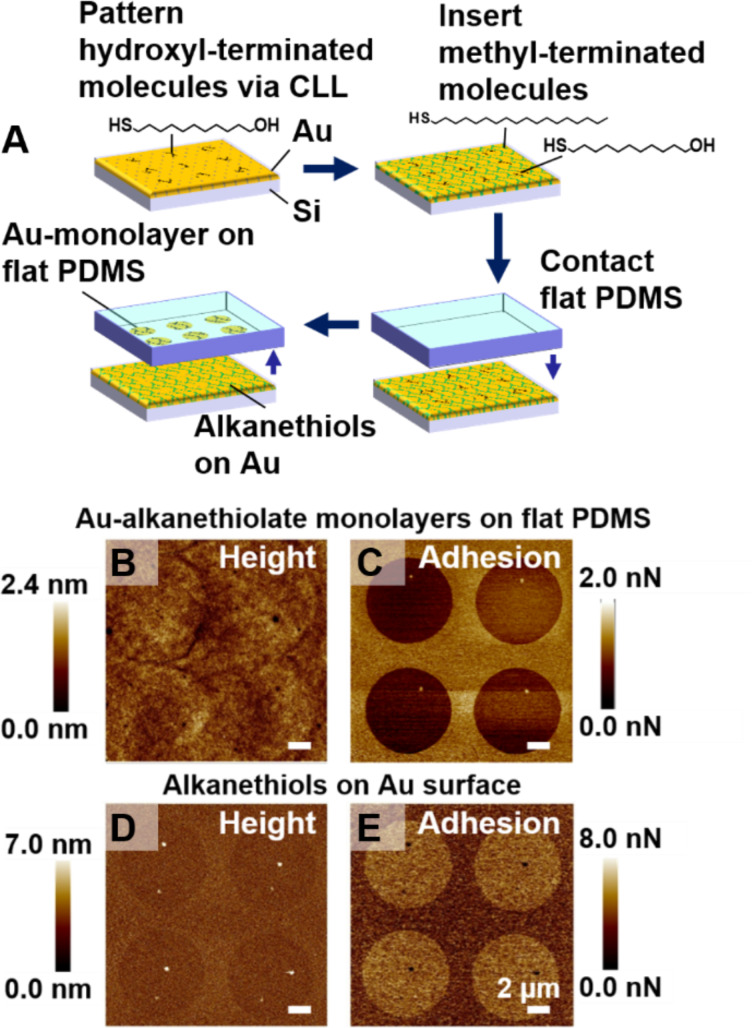
Chemically selective lift-off onto a flat poly(dimethylsiloxane) (PDMS) sample. (A) First, chemical lift-off lithography (CLL) was performed with a stamp having recessed circular features and a preformed self-assembled monolayer of mercaptoundecanol. Methyl-terminated alkanethiol molecules were then inserted into the contact regions, resulting in a self-assembled monolayer with patterned terminal functionalities. Performing a second round of CLL using this substrate and flat PDMS sample resulted in lift-off of the Au–alkanethiolate monolayer from regions containing hydroxyl-terminated molecules. (B) Height and (C) adhesion maps of the Au–alkanethiolate features on PDMS. These maps were simultaneously acquired using peak-force atomic force microscopy. (D) Height and (E) adhesion maps of the remaining alkanethiols on Au after CLL. The topography and adhesion maps in panels B and C show inverted contrast from those in panels D and E, respectively.

Performing CLL with flat PDMS stamps and patterned SAMs having regions of reactive and unreactive molecules on Au was anticipated to yield patterns on PDMS. The scheme in Figure 3A illustrates this concept. First, CLL was performed using stamps with wells of 7.5 µm diameter and mercaptoundecanol SAMs on the Au surfaces, leaving behind SAMs in the circular regions. Octadecanethiol (C18) molecules were then inserted into the contact regions, resulting in patterned monolayers on Au substrates. Octadecanethiol was selected for the study because we hypothesized its chain length would give sufficient contrast in post-CLL AFM imaging and that it would not displace the remaining mercaptoundecanol monolayer in the circular regions (or prevent it from undergoing CLL with a flat stamp) [36–37]. Two-component SAMs on Au having patterned regions distinguished by different terminal groups were then used for a second CLL step involving flat PDMS.

Height maps of post-CLL flat PDMS and the corresponding Au surface shown in Figure 3B and Figure 3D, respectively, had the expected inverted contrast. The regions with Au–mercaptoundecanol monolayers were observed as protruding circles on the flat PDMS, while regions on the Au-on-Si substrate, from which Au complexes were removed, appeared as recessed circles, demonstrating that lift-off occurred in a chemically selective manner.

The X-ray photoelectron spectroscopy (XPS) spectra of patterned PDMS illustrated the presence of Au in the regions predominantly containing mercaptoundecanol (noncontact regions associated with the first CLL step), but also in the contact regions dominated by inserted octadecanethiol. Residual mercaptoundecanol in the contact regions is due to the incomplete removal of molecules during the first CLL step. This partial removal has been used to advantage in fabricating tethered DNA for high-efficiency hybridization [10] and for investigating spin selectivity in electron transport through DNA [12]. Comparing the XPS peak areas suggested that the amount of Au in the lift-off regions is approximately double the surface concentration of Au in the noncontact regions. We note that the contrast in the topographic AFM map of the Au–alkanethiolate monolayers produced via two-component SAMs (Figure 3B) appears lower than that of the monolayers produced via Au-on-Si masters (Figure 1E–H). We attribute the low topographic contrast in the height maps in Figure 3 to the presence of Au–alkanethiolate compounds in all regions of the patterned PDMS.

In addition to topographic height measurements, we used PF-AFM to determine the adhesion force (i.e., the force needed to pull an AFM tip off a surface) to investigate chemical contrast on patterned PDMS [38]. The patterns of circles seen in the AFM adhesion maps in Figure 3C,E are consistent with differential molecular compositions in the lifted-off vs non-lifted-off regions and the chemically selective removal of molecules terminating in hydroxyl groups and not methyl groups during the second lift-off step, whereby patterned PDMS was produced. Collectively, the data in Figure 3 demonstrate a CLL-centered strategy for regional control of chemical composition on flat PDMS supporting materials.

To evaluate the Au–alkanethiol–PDMS hybrid material further, we quantified the apparent heights of the regions containing the lifted-off Au monolayers using AFM topography maps of patterned PDMS (Figure 1E,F,H). To recognize and to differentiate between regions containing Au–mercaptoundecanol monolayers and PDMS-only background regions, we employed a recently developed image analysis algorithm based on Chan–Vese segmentation [39–41]. This algorithm is an enhanced version of a region-based segmentation method that can be used to detect artifacts and differentiates pattern features from topographically uneven backgrounds, which thresholding strategies cannot straightforwardly accomplish [40]. Furthermore, this algorithm minimizes user bias inherent in delineating regions of interest and maximizes the number of image data points considered. Details and demonstrations of our implementation are provided in the Experimental section and in Figures S8–S10 (Supporting Information File 1).

**Figure 4:**
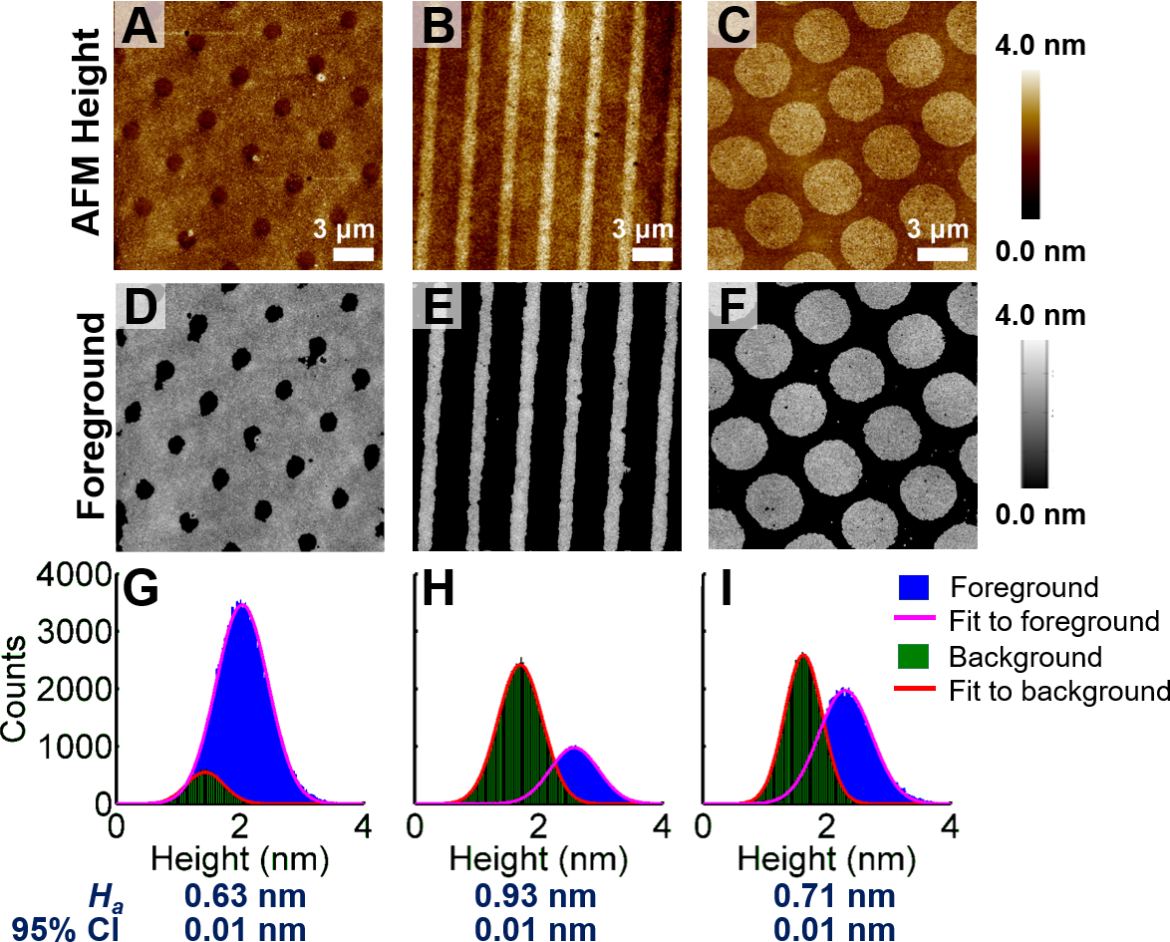
(A–C) Height maps of three different patterns of Au–mercaptoundecanol monolayers on poly(dimethylsiloxane) (PDMS) acquired using peak-force atomic force microscopy. (D–F) Regions classified as “foreground” are determined using the image segmentation algorithm and contain Au–mercaptoundecanol monolayers corresponding to the images shown in panels A–C. (G–I) Histograms of the heights represented by the intensities of foreground and background classifications of pixels. Each histogram was fit to a Gaussian distribution and was consistent with a normal distribution. The calculated apparent height, *H*_a_, determined from each image was the difference in the mean of the foreground and background pixel intensities. The values for *H*_a_ and 95% confidence intervals (95% CI) are shown below each graph.

The Au–mercaptoundecanol monolayers (Figure 4A–C) were associated with heights ranging from 0.63 ± 0.01 nm to 0.93 ± 0.01 nm determined from the Chan–Vese analysis (Figure 4D–I). These apparent heights are smaller than the height of a SAM of mercaptoundecanol on a Au surface, which is 1.3–1.4 nm with a 30° tilt angle relative to the surface normal [42–44]. Considering an interlayer spacing of Au{111} of 2.35 Å [45], the complete lift-off of alkanethiol SAMs from Au surfaces would yield Au–alkanthiol layers approximately 1.6 nm in height, assuming that the molecules retain their original orientation and each thiol removes one Au atom.

Nonetheless, we know that the Au–alkanethiolate monolayers on PDMS resulting from typical CLL experiments are indicative of incomplete lift-off [1,10,31]. Moreover, the dimensions of the Au–alkanethiol complexes that compose the lifted-off monolayer on PDMS are smaller than the spatial resolution of ambient AFM [46]. As such, an average can be calculated for the apparent height by multiplying the typical 60–70% yield of CLL with the full Au–alkanethiolate monolayer height calculated above. Doing so yields an apparent height range of 0.96–1.12 nm, which is still greater than the measured heights (Figure 4). The Au–alkanethiolate complexes on PDMS are expected to adopt a variety of orientations relative to the surface, similar to the variety of orientations of self-assembled alkanethiols at incomplete coverage on Au surfaces [47–48], further reducing our estimate of the apparent height. In all, our measured estimate of the topographic height of a Au–alkanethiol monolayer on PDMS is consistent with all previous CLL characterization attempts and with the predicted one or two atoms lifted-off per alkanethiolate molecule (vide infra).

**Figure 5:**
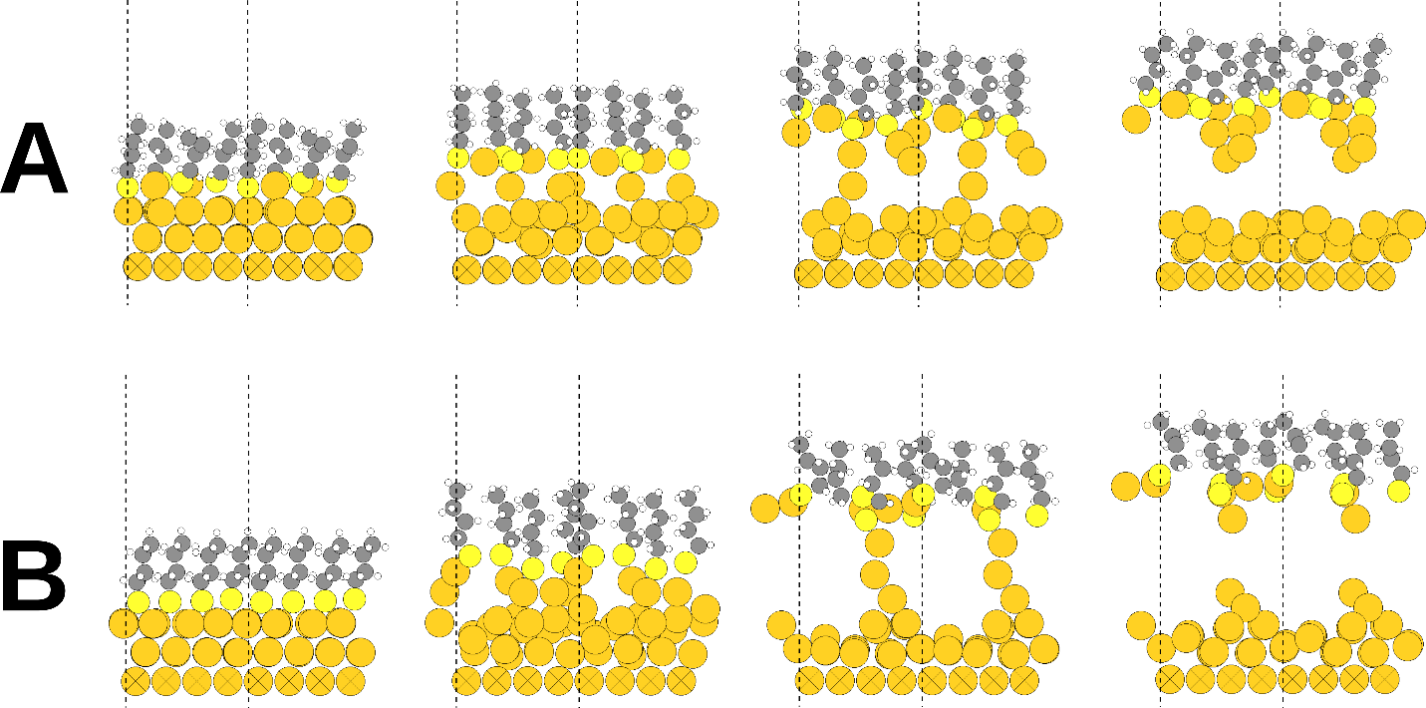
Two configurations calculated by molecular dynamics simulations of lift-off of a butanethiolate SAM on Au{111}. (A) Initially, densely packed RS–Au–SR (R = butyl) units occur on Au{111} having surface vacancies. The number of vacancies equals the number of RS–Au–SR units. (B) Initially, a dense packing of individual butane thiolates occurs at the face-centered cubic sites of a defect-free Au{111} surface. The dashed vertical lines define the borders of each computational unit cell, i.e., in the figure there are two unit cells side by side in each configuration. Atom colors: hydrogen (white); carbon (gray); sulfur (yellow); Au (orange).

The assumptions made above regarding the structure of Au–alkanethiolate monolayers on PDMS are in agreement with estimates of the stoichiometry of the Au–alkanethiolate monolayer calculated through molecular dynamics simulations. Atomic rearrangement during the CLL process was modeled using density functional theory and the grid-based projector-augmented wave (GPAW) method [49]. The simulations revealed that a densely packed SAM of chemisorbed butanethiolates was pulled from a Au{111} surface. The details of the initial Au–thiolate surface structure and the pulling speed were varied (see Experimental section). Figure 5 shows the initial structures and later snapshots from two representative simulations. Figure 5A shows the initial structure having RS–Au–SR units (where “R” refers to the butyl chain) on top of a Au{111} surface with defects, while Figure 5B indicates a close-packed layer of butanethiolates on an ideal fcc Au{111} surface.

During lift-off, some Au surface atoms remain attached to the lifting sulfur atoms, breaking the Au surface symmetry and causing reconstruction of the remaining Au surface layer. As lifting continues, some Au atoms move between the sulfur atoms, forming RS–Au–SR structures that are still able to bond to additional Au atoms. Before complete separation, a chain consisting of two or three Au atoms between each thiol and the Au surface is formed and finally ruptures, usually after the first or second Au atom has attached to each sulfur atom. As a consequence of lift-off, a limited number of Au atoms remain bonded to the lifted thiolate layer, forming a Au–thiol complex with a stoichiometry of up to 1.5 Au atoms per thiol. This stoichiometry corresponds to the removal of 50% of the outermost Au{111} layer bearing a densely packed alkanethiol SAM.

We further computationally analyzed the XPS core-level shifts (CLSs) for each Au atom in the lifted-off complexes (Figure S11, Supporting Information File 1). These calculated spectra are signatures of the predicted structures resulting from CLL of SAMs packed on Au with and without defects. When comparing the spectra and the structures, we found that the shifts are spread ≈1.5 eV around the bulk reference value, and similar chemical environments of the Au atoms resulted in similar core-level shift energies. These simulations indicate that the CLSs of a Au atom in a Au–alkanethiolate monolayer are sensitive to its local environment in the system and that spectral features would reflect the arrangement of self-assembled molecules on the gold surface at initial and/or intermediate stages of CLL. Our current observations are consistent with the predicted stoichiometries, and these simulations form the basis of work to interrogate the structure and stoichiometry of the lifted-off Au monolayer further.

The potential to lift-off Au via PDMS contact is consistent with the discovery that Au–thiolate complexes are the mobile species in SAM diffusion [2,50]. The electronegative sulfur atoms (thiol head groups) withdraw charge from Au atoms, causing measurable changes in the physical properties of Au, including the increased binding energy of Au 4f electrons measured by XPS [51], decreased Au–Au rupture forces in molecular break-junction experiments [52–54], and shorter Au–S bonds compared with Au–Au bonds measured by electron diffraction [55–56]. At molecular resolution, scanning probe measurements have revealed the rearrangement of Au surface atoms [57–59], diffusion and alignment of adatom–adsorbate complexes [50,60], and phase separation of SAMs composed of molecules with different backbones or terminal functionalities [61–63]. Phase separation is driven by stronger intermolecular interactions between one type of SAM molecule vs another in mixed SAMs. The rearrangement and displacement of molecules in mixed monolayers can also be manipulated by choosing other head groups, such as selenols, in place of thiols [64–65].

Theorists have investigated the influence of collective interactions among alkanethiol backbones on the removal of clusters of SAM molecules from Au surfaces [66–67]. For example, less nanomechanical force is required to pull a monolayer of heptanethiolates and Au atoms from a Au substrate than a monolayer of propanethiolates. In addition to previously demonstrated lift-off “compatible” and “incompatible” terminal groups [10–11], the head groups and backbones of the SAM molecules themselves are potential parameters for customizing the composition and chemical state of the lifted-off Au monolayers.

## Conclusion

We have devised a suite of fabrication, imaging, and computation strategies to address the structure, functionality, and stoichiometry of Au monolayers lifted-off during chemical lift-off lithography and we have demonstrated a new 2D Au hybrid material with unique properties. Using CLL, we produced a functional hybrid material of Au–alkanethiolate monolayers on topographically flat PDMS that spatially encodes chemical functionality at the surface of PDMS, while preserving the transparency and flexibility of the PDMS. The lateral dimensions and periodicity of the lifted-off monolayers were preserved from the Au-on-Si masters when patterning the lifted-off monolayers on PDMS, as determined by AFM and SEM imaging. These patterns of Au monolayers were recognizable in fluorescence microscopy when functionalized with thiolated DNA that was hybridized with dye-labeled complementary DNA.

The analysis of the relative heights from AFM images revealed that less than a complete monolayer of Au–alkanethiolates remains on the PDMS material, which is consistent with previous findings and indicates that CLL removes ≈70% of molecules from contact regions. In agreement with indirect evidence that a monolayer of Au is removed during CLL, molecular dynamics simulations converged on a stoichiometry of ≤1.5 Au atoms per thiol. These simulations also demonstrate that the lifted-off Au atoms are in an environment distinct from that at the surface of the bulk Au and are predicted to be distinguishable in photoelectron spectra.

This body of evidence demonstrates that CLL, an already straightforward method for patterning square centimeter areas of alkanethiol monolayers of Au-on-Si substrates, can also be used to pattern PDMS with Au and to impart encoded chemical functionality without affecting the flexibility or transparency of PDMS. Incorporating chemical functionality onto PDMS will be useful for integrating sensing functions into microfluidic devices [68–80]. Compared with many techniques used to impart sub-micrometer features onto PDMS [74–76], CLL is parallel, high-throughput, and is performed under ambient conditions.

Further studies will test the impact of the composition of the supporting molecules on the properties of the lifted-off Au monolayer. The structural and electronic properties of the Au monolayer can be tailored by varying the properties of the supporting molecules [2]. For example, limiting the degrees of freedom of the supporting monolayer by replacing mercaptoundecanol with unsaturated alkanethiols or rigid cage molecules may result in a monolayer that better maintains a planar two-dimensional geometry [2]. Additionally, carboranethiols, which are known to form pristine and nearly defect-free SAMs [61,81–82], or molecules with additional interactions among the backbones, such as 3-mercapto-*N*-nonylpropionamide, which forms hydrogen-bonding networks [83–84], are hypothesized to increase the yield of lifted-off molecules during CLL and to produce more intact, supported monolayers [2]. Replacing thiol moieties with groups that bind more strongly to Au [4,64–65], such as selenolates, will also be investigated. Thus, a rich variety of tunable variables, including stamp geometry, chemical backbone, and anchor groups remain to be explored for CLL.

Ultimately, the resolution of CLL will be defined by the ability to control the separation between individual lifted-off Au–alkanethiolate regions on PDMS (or other supports). It may be possible to dilute the “liftable” alkanethiols on gold [8] to reach the ultimate limit of lifting off single molecules. However, the fidelity of the features achieved (i.e., the ability to replicate features defined by the alkanethiol monolayers on Au onto the PDMS) will increase with increasing CLL yield. In addition, increasing the CLL yield will improve the fidelity of the patterns of Au–alkanethiols lifted-off on PDMS, and thus, presumably more complete and closely packed supported Au monolayers on PDMS.

The computation, fabrication, and visualization strategies established herein form a basic toolbox for interrogating the influence of these variables on CLL and the structure and functionality of the resulting hybrid materials. Further development of CLL has significant potential for fabricating sensors, biocompatible platforms, and other applications that will benefit from flexible, transparent, bio-inert materials combined with the extensive functionalization chemistries of Au.

## Experimental

### Fabricating patterned polydimethylsiloxane stamps

Stamps with topographic features were prepared as previously described [85]. The Sylgard^®^ 184 silicone elastomer kits were purchased from Dow Corning (Midland, MI, USA). The elastomer base and curing agent were mixed in a 10:1 ratio by weight, stirred for 3–5 min, and degassed in a vacuum desiccator for at least 1 h to remove air bubbles. Degassed mixtures were poured over silicon molds (purchased from KTek Nanotechnology, LLC, Wilsonville, OR, USA or fabricated by photolithography) situated in Petri dishes. After degassing again, the PDMS stamps were cured in an oven at 60 °C for 12 h. The PDMS stamps were separated from the silicon masters carefully and cut into desired sizes.

### Patterning Au-on-silicon masters

Silicon wafers with 100 nm Au and 5 nm titanium adhesion layers (Platypus, Madison, WI, USA) were trimmed with a diamond scribe to ≈1 × 1 cm sample size. The substrates were annealed with a hydrogen flame and incubated in 1.0 mM ethanolic solutions of mercaptoundecanol overnight at room temperature and ambient pressure to form SAMs. The patterned PDMS stamps were treated with oxygen plasma (Harrick, Ithaca, NY, USA) for 40 s and contacted with SAMs. The stamps were removed from Au substrates after 2 h. The substrates were then treated with 20 mM iron(III) nitrate and 30 mM thiourea for 10–15 min to etch the Au selectively from the exposed regions.

### Fabricating flat poly(dimethylsiloxane) stamps

The PDMS stamps were templated using featureless silicon wafers. The silicon wafer pieces (Silicon Quest International, San Jose, CA, USA) were degreased by sonicating sequentially for 5 min in ethanol, 3 min in deionized water, and 5 min in ethanol. The silicon wafer pieces were immediately rinsed with ethanol and blown dry with compressed nitrogen gas. They were then exposed to hexamethyldisilazane vapor for 10 min in a closed chamber to facilitate later removal of PDMS. Glass slides (VWR, Radnor, PA, USA) were trimmed to ≈2.5 × 2.5 cm squares and sonicated for 20 min in 1% (w/v) Alconox, rinsed with deionized water, and cleaned two additional times. Clean glass pieces were stored in deionized water until they were rinsed and blown dry immediately before use.

Using a plastic spatula with a tapered tip, 1–2 drops of degassed PDMS (10:2 elastomer/curing agent by weight) were placed on the silicon pieces and degassed for an additional 5–10 min. Flat PDMS films were physically attached to glass slide pieces to minimize damage to their surfaces during handling. Dry glass slide pieces were treated with an oxygen plasma for 40 s. Upon removing the silicon pieces with PDMS from the desiccator, a small drop of PDMS was placed on each glass slide, which was then placed gently on top of the PDMS. The “sandwiches” were cured on a hot plate at 110 °C under a 4.5 kg steel-brick weight. After 10 min, the heat was turned off while the “sandwiches” remained under the weight overnight.

### Patterning flat poly(dimethylsiloxane) stamps

The patterned Au-on-Si masters were annealed with a hydrogen flame and then immersed in 1.0 mM mercaptoundecanol overnight to form new SAMs on the patterned Au regions. Prior to performing CLL, the masters were sonicated three times for 1 s in fresh ethanol, rinsed, and blown dry. The PDMS on glass pieces was removed from the silicon templates immediately before use, rinsed with ethanol, blown dry with compressed nitrogen, and O_2_-plasma-treated for 40 s to activate surfaces. The Au-on-Si masters were placed face down on the PDMS samples. After initial contact and gently pressing by hand, no additional vertical pressure was applied. The contacted regions were lightly marked on the glass underside with a permanent marker. After contact for 2–24 h, depending on the experiment, the Au-on-Si masters were carefully removed from the PDMS. The marked regions were scratched lightly into the PDMS before each sample was rinsed on both sides with ethanol and blown dry.

### Peak-force atomic force microscopy

A Bruker Dimension Icon scanning probe microscope (Bruker Nano, Santa Barbara, CA, USA) was used to map the topography and mechanical properties of flat PDMS stamps patterned with Au–alkanethiolate monolayers. The AFM images of the PDMS stamps (flat and patterned) were measured using the peak force quantitative nanomechanical property mapping mode. ScanAsyst-Air cantilevers (Bruker, spring constant = 0.4 ± 0.1 N/m) were calibrated with a clean piece of silicon before each measurement. A peak-force set-point between 200 and 400 pN was maintained, except where otherwise indicated. These conditions enabled sufficient contact between tips and samples for imaging, while minimizing the load from the cantilever applied to the PDMS.

### Scanning electron microscopy of Au-on-Si masters

Scanning electron microscopy was performed using a JEOL JSM-6700F scanning electron microscope (JEOL, Inc., Tokyo, Japan) with a 750 V DC detector bias and 5 kV accelerating voltage.

### Field-emission gun variable pressure electron microscopy of Au on PDMS

The scanning electron micrographs of Au–alkanethiolate monolayers on flat PDMS were imaged with a low-vacuum detector in a Nova NanoSEM 230 microscope (FEI, Czech Republic) operating at an accelerating voltage of 5 kV. The samples were affixed to the SEM stub and grounded by conductive carbon and copper tape. Variable pressure SEM (VP-SEM) was performed under 50 Pa of water vapor in the sample chamber to avoid charging of the insulating PDMS surfaces by the electron beam.

### Functional DNA patterns on supported Au monolayers

As-received DNA (Integrated DNA Technologies, Coralville, IA, USA) was diluted in nuclease-free water (QIAGEN, Valencia, CA, USA) to make 100 µM stock solutions. Immediately prior to experiments, the DNA stock solutions were diluted 1:100 with 1× phosphate buffered saline (PBS) ([NaCl] = 138 mM, [KCl] = 2.7 mM, and [MgCl_2_] = 5 mM) pH 7.4 (Sigma-Aldrich, St. Louis, MO, USA) to make 1 µM solutions. The patterns of Au–alkanethiolate monolayers on flat PDMS substrates were functionalized with thiolated single-stranded DNA solutions by pipetting 50–100 µL of 1 µM DNA solutions onto the substrates to cover the patterned regions and incubating for ≈20 h at room temperature. The substrates were then thoroughly rinsed with deionized water and blown dry with nitrogen gas. For DNA hybridization on Au–alkanethiolate monolayers on flat PDMS, 50–100 µL of 1 µM AlexaFluor^®^ 488-labeled complementary DNA was pipetted onto the substrates, which were then incubated for 1 h at room temperature. During incubation, the substrates were kept in the dark to minimize photobleaching of fluorescent dyes by ambient light. The substrates were rinsed again with deionized water and blown dry with nitrogen gas.

The DNA duplexes on Au–alkanethiolate monolayers were imaged at an emission wavelength of 517 nm (AlexaFluor^®^ 488; excitation at 492 nm) with an inverted fluorescence microscope (Model: Axio Observer.D1) equipped with an AxioCam MRm charged-coupled device camera (Carl Zeiss MicroImaging, Inc., Thornwood, NY, USA) and a fluorescence filter with excitation and emission wavelengths at 470 ± 20 nm and 525 ± 25 nm, respectively (38 HE/high efficiency, Carl Zeiss MicroImaging, Inc.).

In Figure 2A,B, the patterns were formed on an unsupported slab of PDMS. These patterns were functionalized with thiolated single-stranded DNA (5′-TCT CAA GAA TCG GCA TTA GCT CAA CTG TCA ACT CCT CTT T/3ThioMC3-D/-3′) using the procedure described above. Thiolated DNA strands were hybridized with dye-labeled complementary strands (5′-AAA GAG GAG TTG ACA GTT GAG CTA ATG CCG ATT CTT GAG A/3AlexF488N/-3′). The samples were then imaged with the patterned side facing down in a drop of deionized water on a clean cover slip. The magnification and exposure time was adjusted appropriately for each patterned region. The same preparation and imaging strategy was employed for samples in Figure S5, Supporting Information File 1.

For patterns in Figure 2C,D, the samples were prepared on thin PDMS substrates supported on glass, similar to the sample shown in the photograph in Figure S4, Supporting Information File 1. Thiolated DNA and dye-labeled complementary sequences were 5′-/5-thioMC6-D/ GCA CGA AAC CCA AAC CTG ACC TAA CCA ACG TGC T-3′ and 5′-/5-Alex488N/ AGC ACG TTG GTT AGG TCA GGT TTG GGT TTC GTG C-3′. For control experiments, substrates functionalized with thiolated DNA were incubated with 1 µM AlexaFluor^®^ 488-labeled fully scrambled DNA sequences (5′-/5-Alex488N/ CAT GAA CCA ACC CAA GTC AAC GCA AAC GCA TCA A-3′) to test the specificity of DNA hybridization on patterns of Au–alkanethiolate monolayers. In other experiments, the substrates were incubated with 1× PBS pH 7.4 without thiolated DNA followed by incubation of 1 µM AlexaFluor^®^ 488-labeled complementary DNA. Each substrate was positioned on the microscope sample holder such that the PDMS side was facing away from the light source and the rear side (glass side) of the substrate was facing toward the light source. The images were collected under dry or aqueous conditions. Deionized water drops were pipetted onto the PDMS side of glass substrates to cover the ultrathin Au patterns for imaging under aqueous conditions.

### X-ray photoelectron spectroscopy

The XPS spectra were acquired on an AXIS Ultra DLD instrument (Kratos Analytical Inc., Chestnut Ridge, NY, USA) under ultrahigh vacuum conditions (10^−9^ torr) using a monochromatic Al Kα X-ray source (20 mA, 14 kV) with a 200 μm diameter circular spot size. The pass energy was 80 mV for the survey spectra and 20 mV for high-resolution spectra of the C 1s, S 2p, O 1s, and Au 4f regions. All data points were acquired with a 200 ms dwell time. For adequate signal-to-noise, the number of scans was adjusted for different regions of the spectrum to account for different relative sensitivity factors and low amounts of Au, ranging from 20 scans for C 1s to 100 scans for Au 4f. Because PDMS is an insulator, a charge neutralizer (flood gun) was used to offset charging of the samples that otherwise impedes spectral acquisition. Doing so, however, causes the peak to shift to lower energies as compared to their expected energy obtained without using a flood gun.

### Chan–Vese segmentation

In our implementation, AFM topography maps were segmented into foreground regions, which contained lifted-off complexes, and background regions, which contained only PDMS. The algorithm also output a matrix indicating the location of artifacts, which were then excluded from subsequent analysis of both the foreground and background regions (Figure S8B). During post-segmentation analysis, the histograms of the two regions were plotted and then fit to Gaussian distributions. Because the data were normally distributed, the apparent height of the lifted-off layers was calculated through the difference of the mean of the foreground and background pixel intensities.

Calculating the apparent height line-by-line along the fast-scan direction (a conventional way of calculating the average intensity difference in each line) gave similar values for the apparent height as that calculated using all image pixels (Figure S9, Supporting Information File 1). The imaging force set-point chosen for use in these studies provided sufficient force for imaging, while minimizing the deformation of Au–alkanethiolate monolayers. The apparent height was shown to be equally and minimally influenced by the imaging force (Figure S10, Supporting Information File 1).

### Molecular dynamics simulations

Molecular dynamics simulations were carried out using density functional theory with the Perdew–Burke–Ernzerhof functional [86] using a gridded-based projector augmented wave code [49,87]. In total, 12 pulling simulations were performed using a grid basis (with a grid spacing of 0.2 Å) and a linear combination of atomic orbitals basis with double-zeta polarized functions. The thermal movement of atoms was simulated using the Langevin thermostat targeting room temperature, implemented in the atomic simulation environment [88]. The thermostat adds both a small, random contribution to the force on the atoms and a small friction factor that slows them down, aiming for an average total kinetic energy of the atoms that corresponds to the target temperature. The time step for molecular dynamics was 2 fs. To maintain the stability of hydrogen atoms on this time scale, the mass was increased to the mass of deuterium.

The unit cell was orthogonal with a size of 8.87 Å in the *x*-direction and 10.24 Å in the *y*-direction, in which the unit cell was also set to be periodic. In the *z*-direction, a 10 Å vacuum was set both above and below the structure. In the unit cell, the Au slab consisted of (3, 4, 3) atoms in the (*x*, *y*, *z*) directions, respectively, fulfilling a {111} surface structure with the surface vector pointing in the *z*-direction. The lattice constant was 4.18 Å, corresponding to the theoretical lattice constant of Au in the Perdew–Burke–Ernzerhof approximation. Gamma-points were used in each direction. In addition, four 1-butanethiolates were set on the Au surface forming a (3 × 2√3)-rectangular-symmetric structure. Individual thiolates were set to the fcc positions of the Au{111} surface. In the case of the RS–Au–SR units, the Au adatoms were set to bridge positions and the sulfur atoms to positions above the surface and next to adatoms. Butyl was chosen for the alkyl tail as long enough to form the (3 Å ≈ 2√3) rectangular symmetry naturally but short enough to keep computational costs as low as possible [89].

Before removal, the system was heated up to room temperature using the Langevin thermostat with a friction parameter of 0.002 s^−1^; the heating procedure was run for 2 ps in simulated time. The lowest layer of Au was fixed in its initial position to enable the removal of the thiolates. The pulling moved the terminal carbon atoms with constant velocity outward from the Au surface. Typically, a velocity of 0.5 Å/ps was used. The calculation was continued until thiolate/Au complexes had been completely separated from the surface. The Langevin thermostat was used throughout the calculation to maintain the total energy of the system damping to the energy added due to pulling.

Core-level shifts were calculated for the Au atoms in the modeled structures that were removed from surfaces in the simulations. The density functional theory with the PBE functional was used again via GPAW to calculate the energies of the structures. The procedure followed the one used by Grönbeck [90]. After relaxing the removed structure to a local energy minimum with residual forces below 0.05 eV/Å on any atom, an electron was removed from the 4f core of a Au atom and the change in the total energy of the system was calculated. To make the results comparable, the energy shift of a bulk Au atom was then subtracted from this energy change.

## Supporting Information

File 1 Additional figures.
Details: The supporting information contains additional figures detailing the fabrication of the Au-on-Si masters, unmodified VP-SEM images, reusability, optical, stability, and AFM imaging force studies, fluorescence control experiments, image segmentation details, and computational core-level shift spectra.
