# Supplementary material for: Patterning of supported gold monolayers via chemical lift-off lithography
Source: Beilstein J Nanotechnol. 2017 Dec 8;8:2648–61. doi: 10.3762/bjnano.8.265 (PMC5727779; doi:10.3762/bjnano.8.265)
Supplement: File 1 — Additional figures. Details: The supporting information contains additional figures detailing the fabrication of the Au-on-Si masters, unmodified VP-SEM images, reusability, optical, stability, and AFM imaging force studies, fluorescence control experiments, image segmentation details, and computational core-level shift spectra. [file Beilstein_J_Nanotechnol-08-2648-s001.pdf]

Supporting Information

for

Patterning supported gold monolayers via chemical lift-off lithography

Liane S. Slaughter[§1,2], Kevin M. Cheung[1,2], Sami Kaappa[3], Huan H. Cao[1,2], Qing Yang[1,2], Thomas D. Young[1,2], Andrew C. Serino[1,4], Sami Malola[3], Jana M. Olson[5], Stephan Link[5,6], Hannu Häkkinen*[3,7], Anne M. Andrews*[1,2,8] and Paul S. Weiss*[1,2,4]

Address: [1]California NanoSystems Institute, University of California, Los Angeles, Los Angeles, CA 90095, USA; [2]Department of Chemistry and Biochemistry, University of California, Los Angeles, Los Angeles, CA 90095, USA; [3]Department of Physics, Nanoscience Center, University of Jyväskylä, FI-40014 Jyväskylä, Finland; [4]Department of Materials Science and Engineering, University of California, Los Angeles, Los Angeles, CA 90095, USA; [5]Department of Chemistry, Rice University, Houston, Texas, 77005, USA; [6]Department of Electrical and Computer Engineering, Rice University, Houston, Texas, 77005, USA; [7]Department of Chemistry, Nanoscience Center, University of Jyväskylä, FI-40014 Jyväskylä, Finland and [8]Department of Psychiatry and Biobehavioral Sciences, Semel Institute for Neuroscience and Human Behavior, and Hatos Center for Neuropharmacology, University of California, Los Angeles, Los Angeles, CA 90095, USA

Email: Paul S. Weiss* - psw@cnsi.ucla.edu; Anne M. Andrews* - aandrews@mednet.ucla.edu;
Hannu Häkkinen* - hannu.j.hakkinen@jyu.fi

* Corresponding author

§ Current address: Division of Life Science and Institute for Advanced Study, The Hong Kong University of Science and Technology

Additional figures

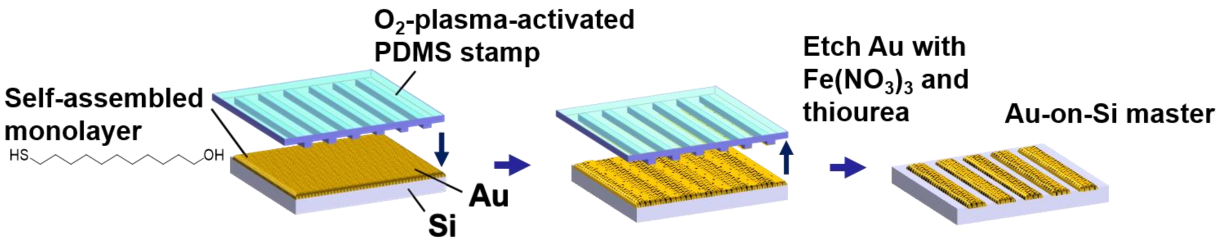

Figure S1: Chemical lift-off lithography (CLL) to produce patterned Au-on-Si masters. Topographically patterned polydimethylsiloxane (PDMS) stamps were used to pattern alkanethiol self-assembled monolayers (SAMs) on Au substrates [1]. Alkanethiols were removed from stamp-substrate contact regions as a result of the lift-off process. Solutions of $Fe(NO_3)_3$ and thiourea were then used to remove the exposed Au from the contact regions. The closely packed SAMs remaining in the non-contact regions acted as molecular resists protecting the underlying Au from etching. The resulting Au-on-Si masters were used to pattern flat PDMS, as shown in Figure 1.

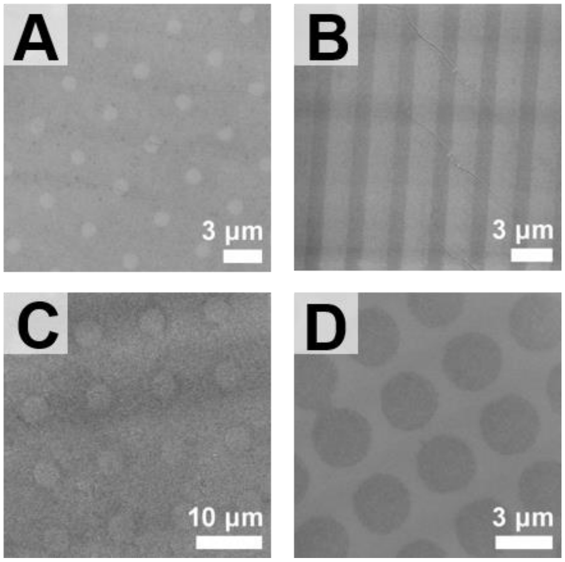

Figure S2: (A-D) Unmodified variable-pressure scanning electron micrographs of Au-alkanethiol patterns corresponding to Figure 1(I-L), respectively.

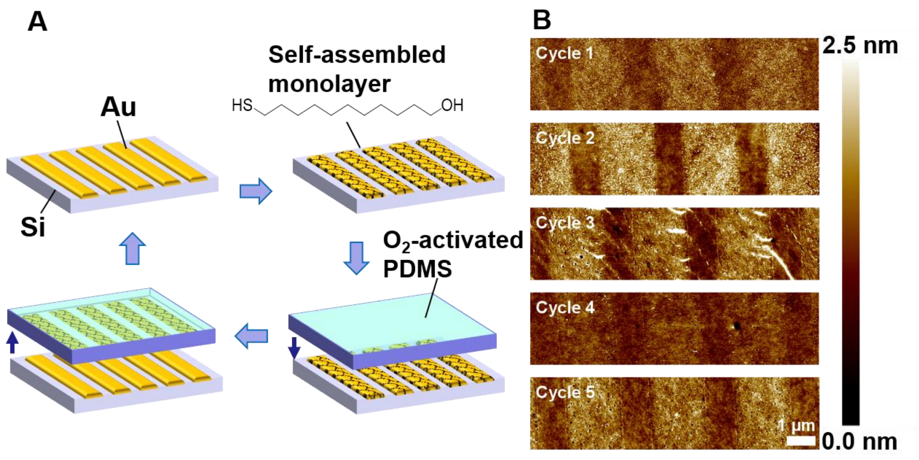

Figure S3: Reuse of Au-on-Si masters to pattern multiple polydimethylsiloxane (PDMS) substrates. A) A patterned Au-on-Si master was functionalized with mercaptoundecanol and used to pattern PDMS by chemical lift-off lithography. After patterning the first PDMS sample, the Au-on-Si master was reannealed, functionalized with a new self-assembled mercaptoundecanol monolayer, and used for lift-off lithography to pattern a second PDMS sample. B) Atomic force microscopy height maps of patterned Au-mercaptoundecanol monolayers on PDMS after five consecutive patterning cycles using the same Au-on-Si master.

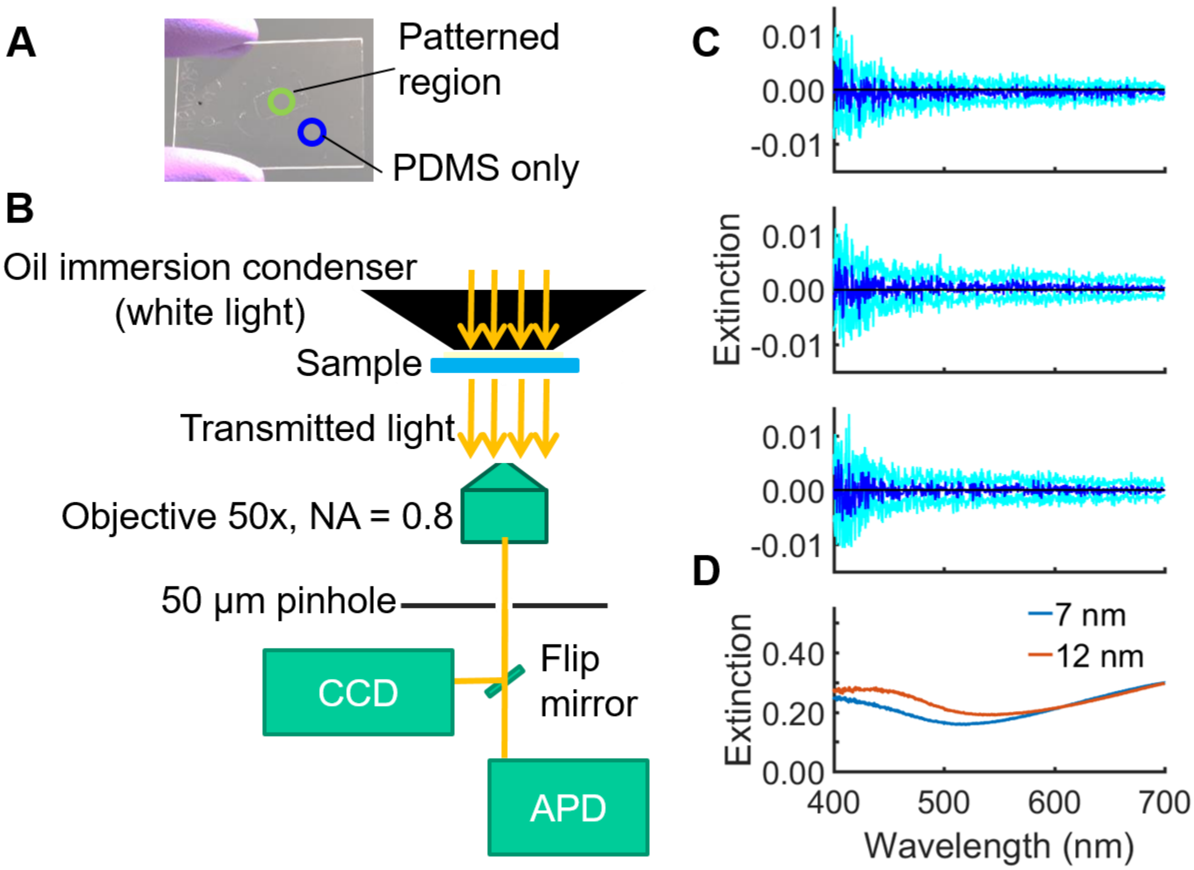

Figure S4: Optical extinction spectroscopy of lifted-off monolayers. A) Photograph of PDMS indicating a region containing a lifted-off monolayer. B) Schematic showing the configuration for measuring optical extinction with sub-micron resolution. The locations to collect spectra were selected after acquiring initial images on an avalanche photodiode (APD). The transmitted light was then redirected to a charge-coupled device (CCD) spectrometer. Signals were collected from regions with ($I_{gold\,+\,substrate}$) and without ($I_{substrate}$) lifted-off monolayers and extinctions were calculated using the following equation:

$$Extinction_{gold} = -log\left(\frac{I_{gold+substrate}}{I_{substrate}}\right)$$

C) Representative spectra from three individual ~1-µm diameter areas containing lifted-off Au monolayers. The dark lines are averages of three spectra, while the light lines indicate standard deviation envelopes. D) Extinction spectra of nanothin Au films. Note the range of the y-axis in D compared with ranges in C. The thicknesses of the Au films measured in D were confirmed by atomic force microscopy.

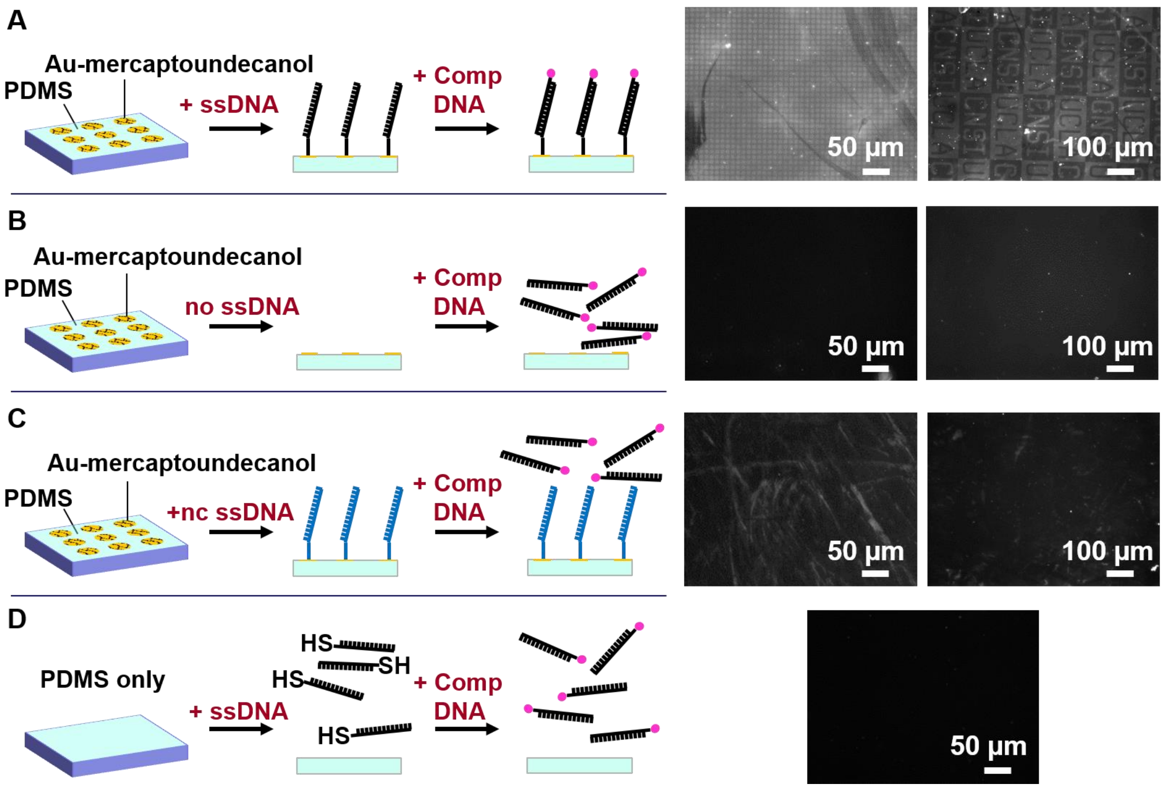

Figure S5: Experiments to test the specificity of DNA hybridization on Au-mercaptoundecanol monolayers. A) Typical substrates with Au-mercaptoundecanol monolayers patterned in circles (left) or CNSI letters/UCLA relief (right) with self-assembled thiolated single-stranded DNA hybridized with fluorescent-dye-labeled complementary sequences. B) Thiolated DNA was omitted. C) Noncomplementary thiolated single-stranded DNA was self-assembled prior to hybridization. D) Unpatterned substrate having no Au-mercaptoundecanol monolayers. The same thiolated DNA and fluorescently-labeled complementary sequences in Figure 2 were used here.

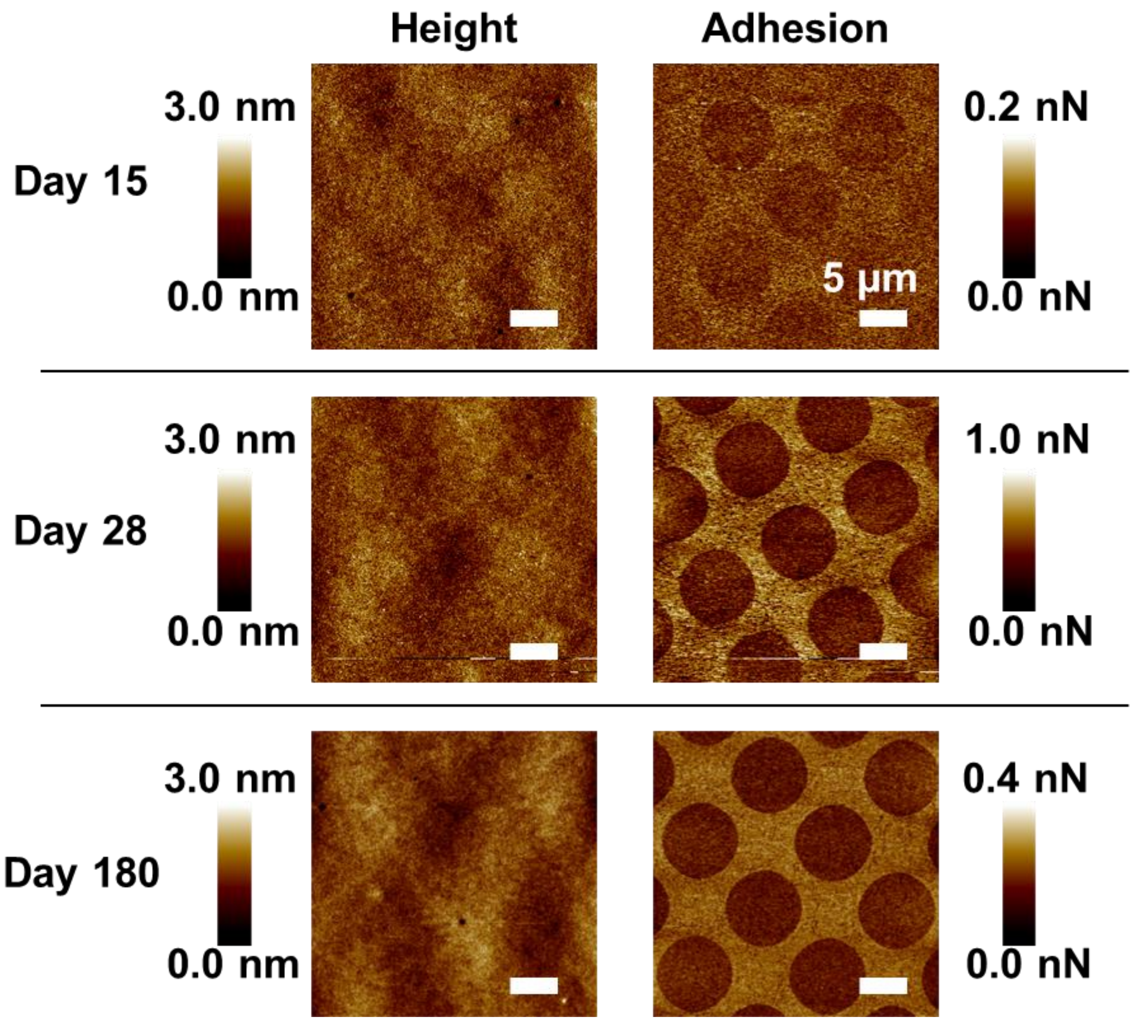

Figure S6: Atomic force micrographs of a Au-alkanethiol monolayer pattern on flat polydimethylsiloxane at 15, 28, and 180 days after sample preparation. The sample was rinsed with ethanol and dried with compressed nitrogen prior to recording each image.

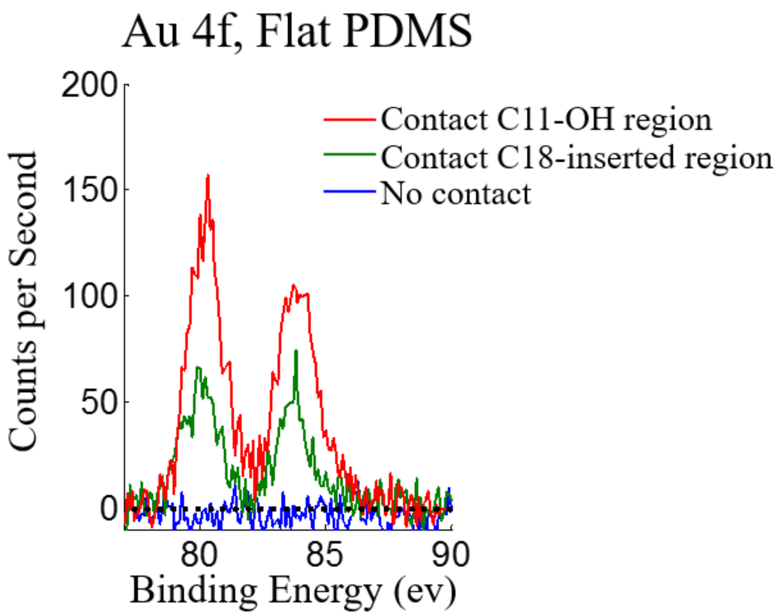

Figure S7: Representitave X-ray photoelectron spectroscopy (XPS) spectra from different regions of flat polydimethylsiloxane (PDMS) contacted with self-assembled monolayer (SAM) molecules on a Au surface patterned by the procedure illustrated in Figure S1. Here, chemical lift-off was performed by contacting an activated flat PDMS stamp with half of each Au substrate having a preformed SAM comprised of mercaptoundecanol (C11-OH). Each substrate was then immersed in a 1 mM ethanolic solution of octadecanethiol (C18) to insert C18 molecules into the contact regions. Then, entire substrates were contacted with new, activated flat PDMS stamps. Each region of the patterned PDMS was analyzed by XPS. Peak areas from the C18 contact regions (green trace) were 46 ± 12% of the peak areas from the C11-OH contact regions (red trace) (averaged across N=4 substrates). These measurements indicate that C11-OH molecules are not completely removed or displaced during lift-off and C18 insertion.

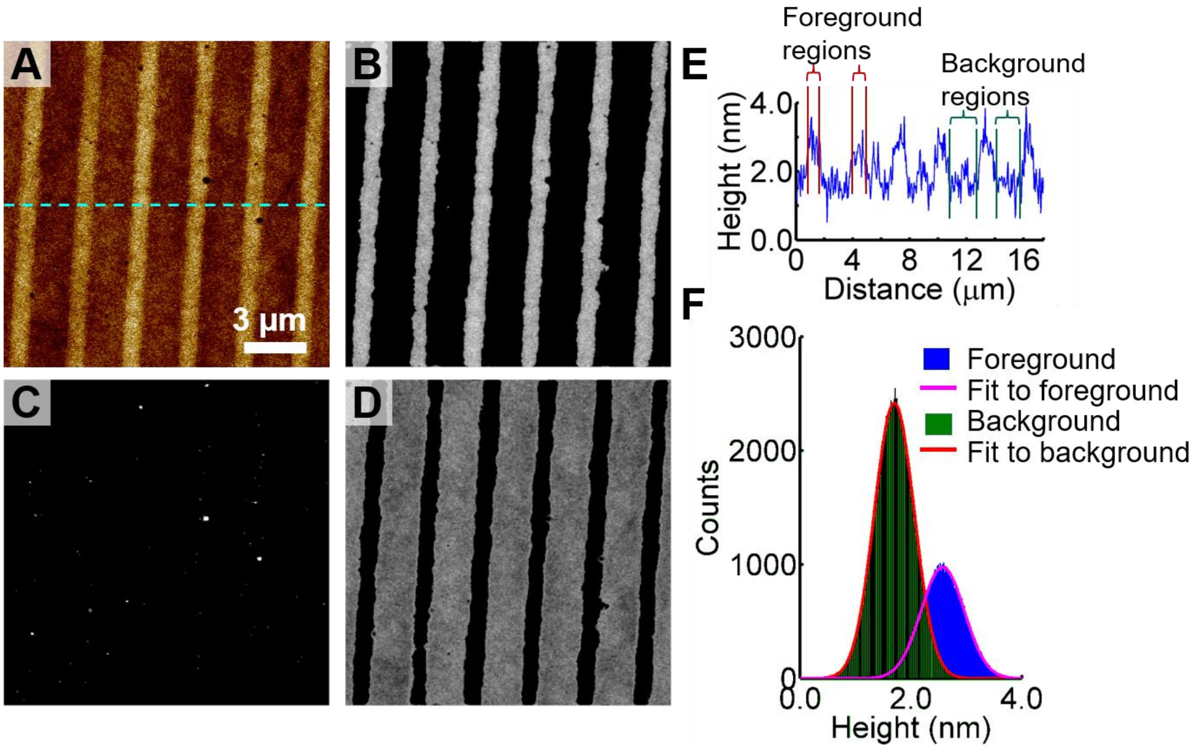

Figure S8: Demonstration of the Chan–Vese segmentation algorithm applied to a height map acquired by atomic force microscopy. A) The recorded height map displayed with false color scale. B) The foreground segment (light regions) identified by the algorithm, excluding the artifacts. C) The artifacts identified by the algorithm. D) The background segment (light regions), excluding artifacts. E) Height profile along the line section drawn across the image in panel A. Red and green lines and brackets indicate examples of foreground regions and background regions, respectively, which are averaged to calculate the intensity difference in the 'line-by-line' approach. F) Histograms of the heights determined from the pixel intensities in the foreground and background segments and Gaussian fits from the 'all-pixels' approach.

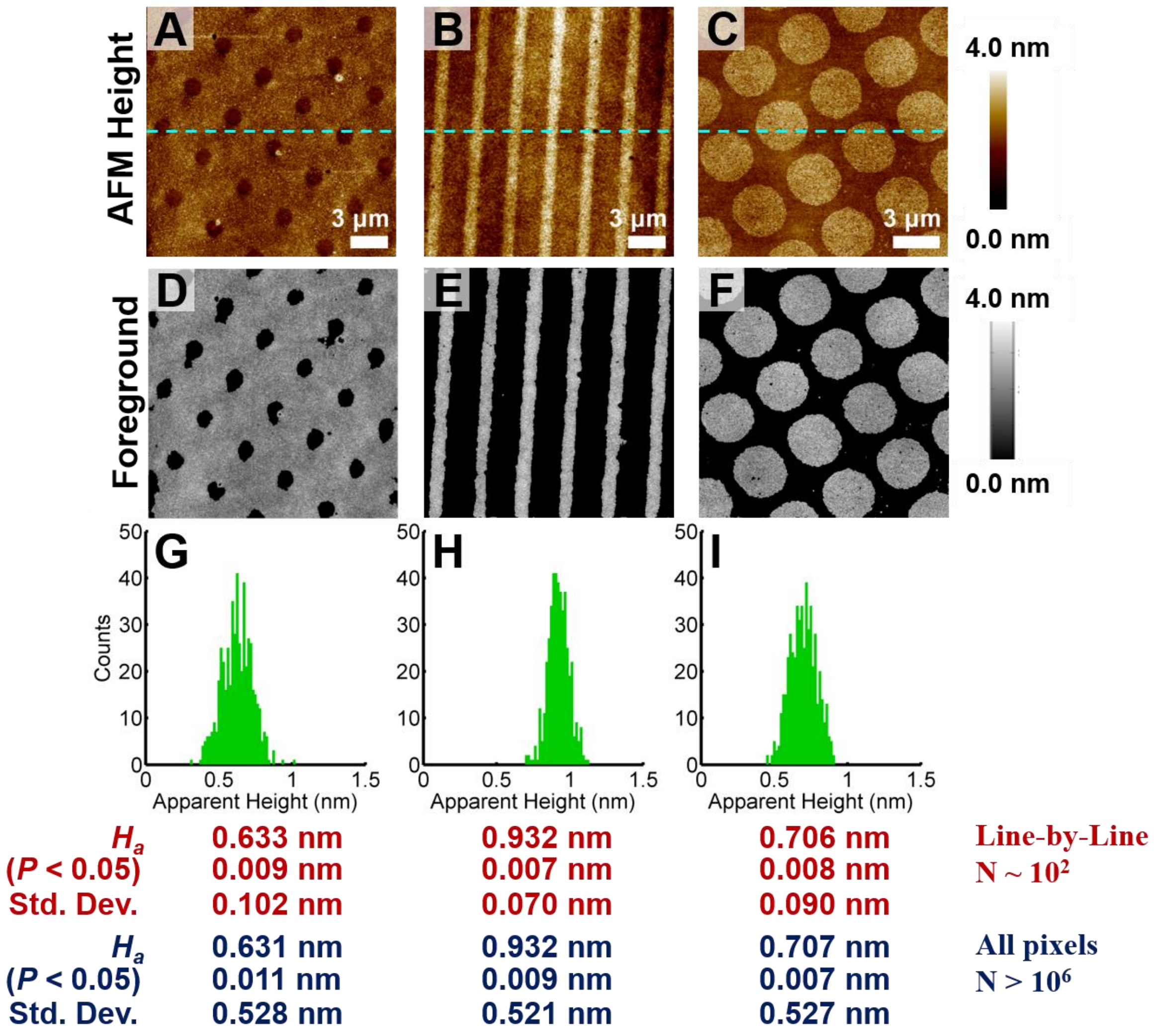

Figure S9: Analyses of apparent heights from atomic force micrographs. (A-C) The same height maps shown in Figure 1 of three different patterns of Au-alkanethiol monolayers on polydimethylsiloxane (PDMS) with representative line segments shown. (D-F) The 'foreground' segments (light regions) containing Au-alkanethiol complexes corresponding to the images in A-C. (G-I) Histograms of the average heights calculated from the differences of the 'foreground' and 'background' heights of each line. Using this approach, the calculated apparent height, H_a, for the foreground segment in each image is the average of the calculated heights from each line. The values for H_a, 95% confidence intervals (95% CI), and standard deviations are indicated below each graph. Values are reported for the line-by-line approach *vs.* the all-pixel approach.

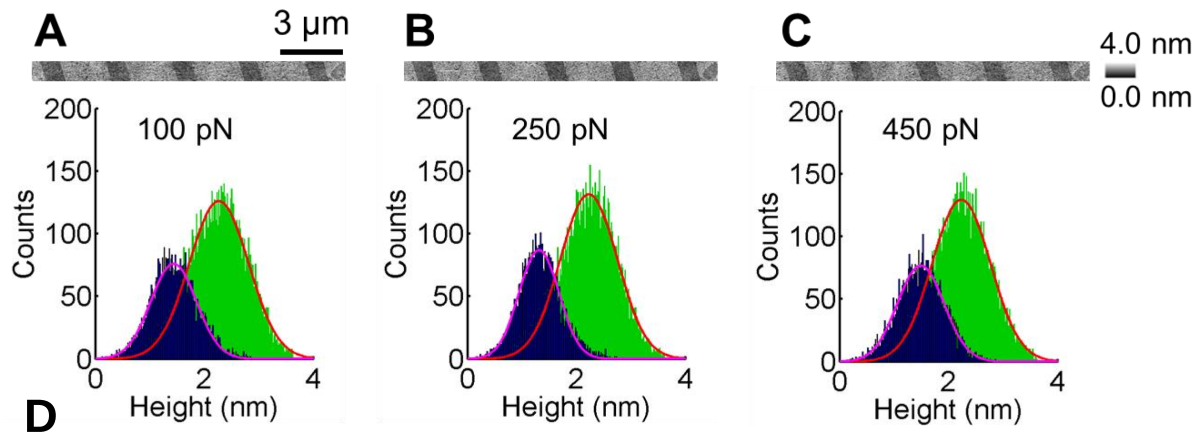

D

Force (pN)	Apparent height (nm)	95% Confidence interval (nm)	HWHM (nm)
100	0.80	0.03	0.49
150	0.88	0.03	0.47
200	0.87	0.03	0.44
250	0.85	0.03	0.44
300	0.80	0.03	0.46
350	0.75	0.03	0.46
400	0.72	0.03	0.46
450	0.74	0.03	0.48

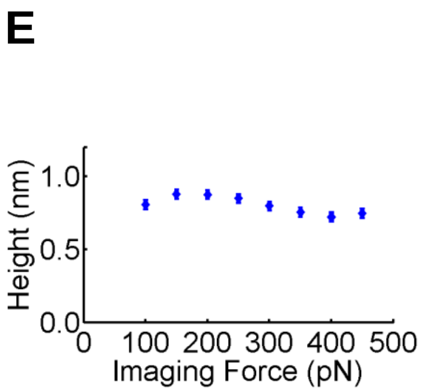

Figure S10: Influence of imaging force on apparent height. (A-C) Height maps of the same 15 μm × 0.93 μm area acquired by atomic force microscopy using the forces indicated on the graphs below. Each graph displays histograms of the heights of the Au-alkanethiol monolayer-containing regions (blue) and the polydimethylsiloxane (PDMS)-only regions (green), as detected by the segmentation algorithm. D) Apparent heights, confidence intervals, and half-widths at half-maxima calculated for each image under different force setpoints. E) Plot of apparent height *vs*. imaging force. Each data point is the apparent height (calculated by Chan-Vese segmentation) ± the confidence interval.

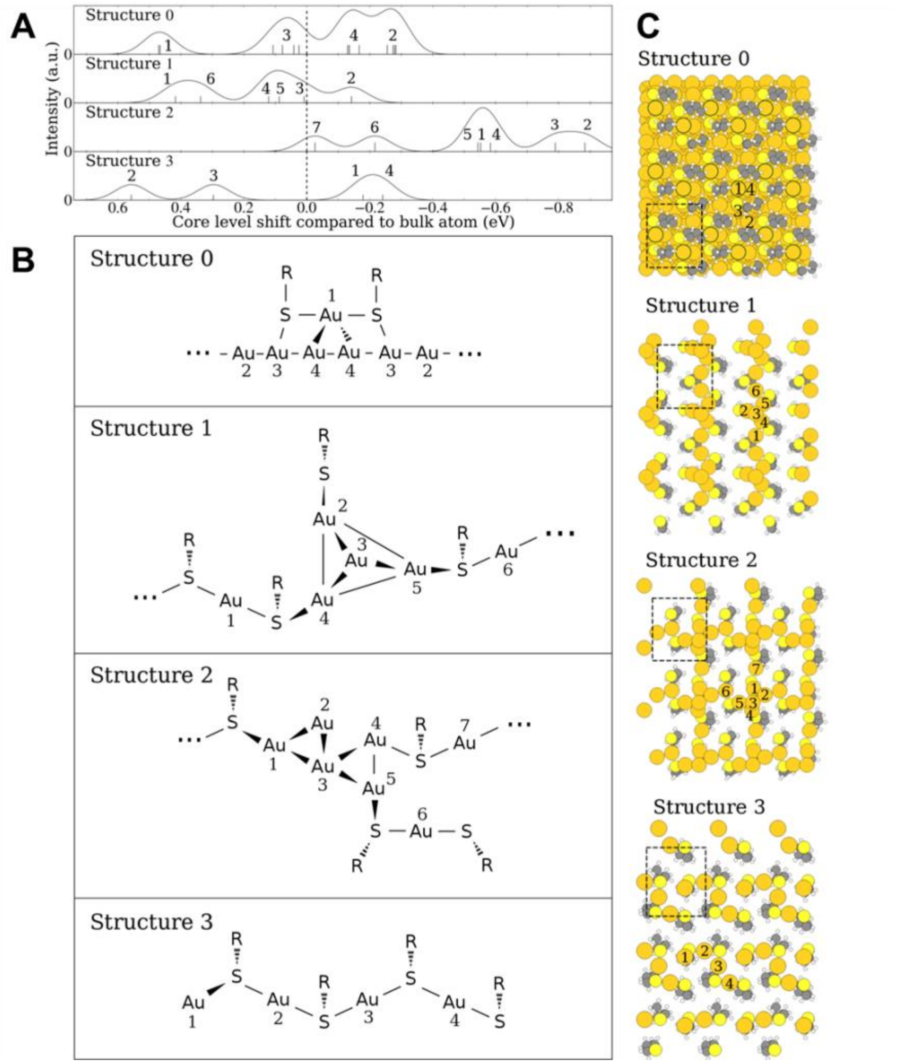

Figure S11: Core-level shift spectra of RS–Au–SR units on Au{111} and three computationally lifted-off Au-alkanethiol complexes and their structures. A) The calculated core-level shift spectra with 0.05 eV Gaussian widening of the peaks. The x-axis represents the energy difference between the shift of an atom in the Au-alkanethiol complex and a bulk atom; a bulk atom is at 0 eV (marked with dashed vertical line). The peaks in the spectra are labeled with numbers corresponding to the Au atoms labeled in the structures of panels B and C. B) Skeletal formulae and indices for Au atoms. The label R refers to butyl chains. The three dots in structures 0, 1, and 2 refer to the periodic complexes. Structure 0 has close-packed RS-Au-SR units on top of an ideal fcc Au{111} surface. Structures 1 and 2 correspond to the removed complexes in the simulations of Figure 6A and B, respectively. Structure 3 originates from another lift-off simulation with similar initial conditions as Figure 6B, however the wave functions were calculated using the double zeta-polarized linear combination of atomic orbitals basis in the grid-based projector augmented wave platform. C) Space-filling structures corresponding to the same structures in B. Structure 0 is shown from above the SAM, while the other structures are shown from below the lifted-off complexes. The dashed rectangles represent the computational unit cell. The circled Au atoms in Structure 0 are adatoms bound between the sulfur atoms.

References

1. Liao, W.-S.; Cheunkar, S.; Cao, H. H.; Bednar, H. R.; Weiss, P. S.; Andrews, A. M. *Science* **2012,** *337*, 1517–1521.
